# Supplementary material for: Are carbon emissions trading and green financial instruments synergistic? -Comprehensive quantitative research based on content analysis
Source: PLoS One. 2024 Mar 7;19(3):e0298601. doi: 10.1371/journal.pone.0298601 (PMC10919601; doi:10.1371/journal.pone.0298601)
Supplement: S1 Data — (ZIP) [file pone.0298601.s001.zip › Data/地级市Z_F工作报告.zip!/╣π╬≈/▒▒║ú╩╨/└┤╫╘╣1⁄2╓┌║┼í╛┬φ┐╦╩2╛▌═°í┐_2018.docx]

各位代表：

现在，我代表北海市人民政府，向大会报告政府工作，请予审议，并请市政协委员和列席会议的同志提出意见。

一、2017年工作回顾

去年四月，习近平总书记视察北海，对北海提出工作要求、寄予殷切期望，为北海加快发展指明了方向、带来了机遇、增添了动力。在自治区党委、政府和市委的正确领导下，在市人大、市政协的大力支持下，我们认真学习贯彻党的十九大精神，以习近平总书记重要指示为指引，凝心聚力、真抓实干、顽强拼搏，深入推进“五个扎实”，大力实施“四大会战”，加快舞起“四个龙头”，完成了市十五届人大二次会议确定的目标任务，保持了经济社会持续较快健康发展的良好势头。

全市经济社会发展和政府工作呈现八个亮点：

一是领跑作用更加凸显。经济发展增速快、质量好、贡献大，继续领跑全区。完成地区生产总值1229.8亿元，增长10.2%，增速居全区第一，是全区唯一实现两位数增长的设区市；财政收入突破200亿元，增长20.1%，增速居全区第一位，实现五年翻一番，对全区财政收入增长贡献率超过20%。

二是工业支撑更加牢固。工业保持两位数增长，对全市经济增长贡献率达50.8%。规模以上工业总产值完成2537.3亿元，增长16.4%，工业产值总量首次提升至全区第四位；规模以上工业增加值完成597.9亿元，增长11.9%。三大主导产业实现总产值2166.3亿元，增长16.7%；主要产业园区完成产值2403亿元，增长16.7%，占全市规模以上工业总产值的94.7%。

三是城乡环境更加优美。城乡面貌发生可喜而实在的变化，市容市貌更加整洁，主城区生活垃圾无害化处理率达100%，城市主干道亮灯率达98%、小街小巷亮灯率超过96%。交通秩序更加规范，“四车”乱象大幅减少。入选第15届中国—东盟博览会中国“魅力之城”，荣获第七轮自治区文明城市，环广西公路自行车世界巡回赛北海赛段比赛精彩安全圆满，展现了靓丽的景观风貌，市民的自信心、自豪感显著增强。近岸海域水质优良率达100%，环境空气质量优良率达92.1%，空气质量综合指数位居全区第一。习近平总书记视察北海时称赞“北海有这么好的空气质量，是群众真正的福利”。

四是开放合作更加深入。北海港口岸正式扩大开放，入选国家海洋经济创新发展示范城市，出口加工区获批国家高新技术产品全球入境维修/再制造示范区和广西首批CEPA先行先试示范基地，国家海洋局第四海洋研究所落户北海，龙港新区和桂台产业合作北海示范区获批设立。

五是改革成效更加显著。一批“接地气”改革举措取得突破，总体投资环境满意度排全区第二。商事制度改革全面推进，“多证合一、一照一码”登记制度改革走在全国前列。成立市行政审批局，划转24个部门的行政许可事项232项，实现“一枚公章管审批”，审批事项平均办结时限由24.2天压缩到6.7天。试行新一轮市级财政管理体制改革，建立广西第一家政府采购电子化交易“政采云”平台。建成“农事网通”政务服务和“阳光红页”信息公开平台，率先在全区实现一网连通基层群众的服务和信息需求。

六是旅游发展更加兴旺。创建国家全域旅游示范区工作全面推进，接待国内旅游者3069.8万人次，增长24.1%；国内旅游消费364.5亿元，增长28.2%。新增4A级景区1家、3A级景区9家，希尔顿逸林、冠岭喜来登等4家国际品牌高星级酒店开工建设。北海机场旅客吞吐量171万人次，增长38.7%，是全国民航机场旅客吞吐量增幅的3倍。进出北海动车车次最多时每天达34对，全年旅客到发量838万人次。

七是民生保障更加有力。全面完成自治区下达的年度脱贫摘帽计划任务和奋斗目标。城乡居民生活水平有新的提高，城镇新增就业2.99万人，农村劳动力转移就业新增1.6万人，城镇登记失业率1.88%，为2004年有准确统计数字以来最低。年初承诺的10件为民办实事事项件件落实，市花公园、体育休闲公园建成开园，中山公园、长青公园升级改造全面启动。北航北海学院办学历史遗留问题妥善处置，市人民医院银滩医院投入使用。

八是社会大局更加稳定。平安北海建设提档进位，群众安全感满意度明显提升，群众对公安机关满意度排全区第一。一批信访难题得到化解，未发生大规模群体性上访事件。以零容忍态度重拳打击传销，侦破传销案件338起，“2·17”特大案创广西传销案件单案逮捕人数之最，传销警情下降50%以上。各类生产安全事故起数下降67.3%，死亡人数下降57.5%，安全生产形势持续稳定向好。

2017年，是全市人民欢欣鼓舞、倍感振奋的一年，是干部群众团结拼搏、攻坚克难的一年，是力补民生短板、解决遗留问题成效明显的一年，是经济社会发展成果丰硕、亮点纷呈的一年。

一年来，我们主要做了八项工作：

（一）着力构筑向海经济新格局。落实习近平总书记关于打造向海经济的重要指示，制定实施一批《工作方案》和《行动方案》，加快编制向海经济发展战略规划，成立北海向海经济研究院。海洋经济规模不断扩大，实现海洋生产总值517亿元，占全市地区生产总值的48%；主要海洋产业实现增加值250亿元，占广西的37.5%，总量排广西第一位。加快振兴南珠产业，实施《关于加快振兴南珠产业的意见》，划定南珠养殖用海专区，启动建设中国南珠产业标准化示范基地，南珠产量和质量双提升。北海港万吨级以上泊位达13个，港口货物吞吐量3190万吨，海运客运量335万人次。

（二）着力推动产业升级发展。坚定不移发展壮大优势产业，完成第一产业增加值190.5亿元，增长3.7%；第二产业增加值668.7亿元，增长10.5%；第三产业增加值370.6亿元，增长13.3%。三次产业对GDP的贡献率分别为6.2%、52.5%、41.3%，产业结构调整为15.5:54.4:30.1。

深入实施工业转型升级大会战，不断推动产业链条完善延伸，着力推进三大主导产业技改扩容增量。电子信息制造业完成产值1323亿元，增长13.5%；石油化工产业完成产值368.6亿元，增长21.9%；临港新材料完成产值474.7亿元，增长22.1%。中石化北海炼化、诚德镍业、惠科、三诺等一批重要企业的引领作用更加明显。工业用电量42.2亿千瓦时，增长20.1%，工业企业用电量与产值增速匹配性较好。

深入实施全域旅游城市建设大会战，出台高星级旅游酒店建设扶持政策，推进旅游景区景点和公共服务体系建设，银滩环境综合整治和合浦县、涠洲岛创建广西特色旅游名县成效明显，涠洲岛南湾鳄鱼山景区列入创5A名单并通过自治区初评。加快推进厕所革命，新建和改建旅游厕所71座。开展“北海遇见北海”“暖冬遇见北海”等媒体、文化、体育与旅游相结合的系列活动，打造复合型城市宣传推介模式。

加快发展商贸等服务业，成功举办“壮族三月三·八桂嘉年华”及电商节北海主会场系列活动，多个城市综合体建成开业，中国电子北部湾信息港、高新区软件和信息服务业基地获批为首批自治区级现代服务业集聚区，凡普金科等知名企业入驻北海。金融生态环境持续向好，在全国首创小微企业“招商贷”金融业务。人民币各项存款余额938.8亿元，增长15.1%；人民币贷款余额654.5亿元，增长22.3%，创历史同期最好水平。第三产业增速位居全区首位，成为拉动全市经济增长的重要力量。

（三）着力加强城市建设管理。深入实施城乡建设管理大会战，入选国家第二批城市设计试点城市，编制一批城市建设与保护重要规划，常住人口城镇化率提高到58%。

加快完善城市基础设施。贵合高速（北海段）建成通车，海洋大道、涠洲岛环岛公路等一批道路竣工通车，冯家江大桥、北海大道（南珠大道-迎宾大道）改造、南珠大道（亚叉岭-西南大道）改造等一批路桥项目开工建设，50条小街小巷完成改造或开工改造，内涝（三期）整治工程顺利实施。铁山港5-6#泊位建成。500千伏变电站等一批电力设施建成投产，全市用电安全性大幅度提升。城市WiFi覆盖项目建成上线试运行，55个公共区域实现免费WiFi覆盖,在广西率先成为城市WiFi覆盖和免费WiFi上网的设区市。

全面整治城乡环境。扎实开展“城乡环境提升年”十大提升行动，全面落实“门前三包”和生活垃圾定点投放定时收集清运制度，开展常态化环境整治夜巡。南珠市场和长青路“严管一条街”改造提升竣工，广东路与茶亭路交叉路口渠化改造和市区23处斑马线改造完成，“黄金北岸”项目启动建设。着力盘活闲置用地、集体就业用地，推进低效工业仓储用地再利用。

大力推进生态建设。制定实施《北海市生态立市行动方案》，严肃整改生态环境突出问题，特别是大力整治中央环保督察组反馈的问题。铁腕整治合浦“天坑”，完成矿坑生态修复，积极推进生态养殖和休闲旅游项目开发。完成全市基本农田划定工作，划定永久基本农田10.6万公顷。实施海域海岛海岸带保护修复工程，划出红线保护岛屿、湿地、红树林、海湾、湖泊，明确规定距海岸线300米范围内禁止新建非公益性永久设施。启动水环境治理三年攻坚战，完成40个雨污合流排放口截流和36个直排口整治，红坎污水处理厂提质达标，全面完成自治区下达的9个乡镇污水处理厂建设任务，主城区生活污水集中处理率达98.5%。开工建设北海生活垃圾焚烧发电等项目。

（四）着力深化改革扩大开放。坚持改革开放、创新驱动，驰而不息落实各项改革措施，加快舞起北部湾经济区扩大开放合作龙头。

持续增强创新能力。制定实施创新驱动发展战略和配套措施。推进专利双倍增计划，每万人发明专利拥有量4件、提升35%。加强创新企业和创新平台建设，新认定高新技术企业23家，新增国家级众创空间2家、自治区级众创空间2家、院士工作站1家、工程技术研究中心1家。北海海洋产业科技园入选国家创新人才培养示范基地，成为广西首个进入国家“万人计划”项目。

全面推进各领域改革。推进“双随机一公开”监管方式改革，企业年报公开率为全区第一。加快推进跨省异地就医直接结算，成为全区第一个实现跨省异地就医直接结算的设区市。城市公立医院综合改革落地见效，告别“以药补医”时代。完成社保费交由地税局征收改革试点任务。农村土地承包经营权确权登记可颁证率达96%，提前完成自治区下达的目标任务。

不断扩大开放合作。加快发展外向型经济，全年招商引资实际到位资金456.9亿元，全口径利用外资4.98亿美元；外贸进出口总额230.9亿元，增长12.5％；加工贸易进出口总额133亿元，增长11.6%。加强区域合作，积极参与北部湾城市群建设，融入北部湾经济区同城化，实现南北钦防四市一卡通互联互通，与玉林市共同推进玉港合作园规划建设。加快培育航空市场，机场一号登机廊桥建成使用，新开通国际航线1条、国内航线10条，平均客座率提高7个百分点。

（五）着力统筹城乡发展。坚持以稳定粮食生产和促进农民增收为导向，加快推进农业农村现代化建设与发展，完成农林牧渔业增加值193亿元，增长3.8%。

扎实推进脱贫攻坚。深入实施农村脱贫攻坚大会战，全面落实精准扶贫政策措施，重点扶持产业增收脱贫和贫困村集体经济发展，多渠道筹措各类帮扶资金11.03亿元，其中市财政预算安排专项扶贫资金1亿多元，是2016年的3倍多。全市46个贫困村、4941户贫困户、2.1万贫困人口达到脱贫摘帽标准，其中21个贫困村、1.7万贫困人口实现脱贫摘帽。

加快发展县域经济。贯彻落实全区县域经济发展大会精神，坚持把产业作为主要抓手，统筹推进县区发展，县区规模以上工业完成产值1156.8亿元，增长21.1%，占全市规模以上工业产值比重45.6%。加大对合浦的财政支持力度，市本级财政共补助合浦县4.3亿元，比2016年增加了一倍多。合浦工业园、铁山东港产业园、月饼小镇建设加快推进，县域产业不断做大做强。合浦县城、特色城镇建设步伐加快，侨港镇入选第二批全国特色小镇培育名录。

稳步发展现代农业。粮食生产保持稳定，完成粮食作物播种面积120万亩。16个农业示范区入选广西现代特色农业示范区，“北海生蚝”成为国家地理标志保护产品。新增农民专业合作社150家、家庭农场61家，市级以上农业产业化龙头企业达35家，农产品质量安全总体较好，重大动物疫病防控工作扎实开展。

深入推进“美丽北海•宜居乡村”建设。大力开展产业富民、服务惠民、基础便民专项活动，为全市29万农（渔）民释放红利，解决2.3万人就近就业，六项农村基本服务覆盖全市行政村，率先在全区超额完成农村改厨改厕工程目标任务，建成12个乡镇垃圾片区处理中心、20个村级垃圾处理中心，建成农村公路372公里，农村生产生活环境明显改善。

（六）着力推进项目建设。加强项目统筹谋划，大力开展招商引资，实施重点项目569个，组织项目集中开竣工11批次，开竣工项目243个。完成固定资产投资1099.7亿元，增长8.8%。

掀起招商引资热潮。开展“商机遇见北海”系列活动，全年“走出去”招商500多批次，接洽来访客商1500多批次、逾6000人次，成功举办佛山不锈钢招商、南京石化招商、郑州商贸旅游招商、深圳IT产业招商等专题招商活动。

健全项目推进保障机制。成立重大项目策划与推进领导小组和七个推进工作组，建立定期会商机制，集中解决项目推进问题。加强项目策划和储备，建立“项目超市”，推行项目与金融机构对接机制，解决项目融资难问题。继续坚持“四定”办法，完善定期督查通报制度，强力推进项目建设。

狠抓产业项目建设。银河城市科技产业城、中电北部湾信息港一期建成开园，斯道拉恩索聚乙烯淋膜生产线、香港云芯、欧米亚微米碳酸钙等一批项目竣工投产，诚德固溶酸洗生产线扩能、北部湾表面处理中心等一批重点项目加快建设，惠科移动智能终端产业链集群项目开工建设。

（七）着力加强社会建设。加快发展社会事业，创新社会治理，持续改善和保障民生，累计完成民生支出124.5亿元，增长6.36%，民生支出占总支出的79%。

大力夯实教育基础。铁山港区顺利通过国家义务教育均衡发展督导评估,合浦县、海城区、银海区通过自治区义务教育均衡发展督导评估。高校在校学生总数突破5万人。建成（迁建）中小学幼儿园6所，新开工建设中小学幼儿园3所。建成市第十一中学，开工建设北京八中北海分校新校区。发放义务教育“两免一补”资金1.6亿元，资助学生11万人次。

繁荣发展文体事业。制定实施《关于加强海上丝绸之路文化遗产保护利用的行动方案》，加快推进海上丝绸之路申遗工作，大型3D动画片《海上丝路南珠宝宝》在中央电视台开播并获广西“五个一工程”奖，《沧海丝路》《北部湾人家》等电视连续剧在北海拍摄，成功举办第二届北海民间文化艺术博览会，新建村级公共服务中心22个。北海体育健儿在第一届北部湾城市运动会上获得总分和金牌数第二名，中国-东盟国际汽车拉力赛在合浦鸣枪开赛，足球、冬泳、自行车等体育活动蓬勃开展。

扎实推进健康北海建设。制定实施《“健康北海2030”规划》，召开全市卫生与健康大会。持续提升公共卫生服务能力，艾滋病防控效果全区最好，北海儿童医院、涠洲岛人民医院、市第二人民医院搬迁工程等项目加快推进。积极引进优质医疗资源，与湘雅医院等多家国内高端医疗机构加强合作。加强食品药品安全监管，大力创建广西食品安全示范城市。

切实保障和改善民生。城市低保对象补助水平由平均每人每月300元提高到330元，农村低保对象补助水平由平均每人每月140元提高到170元。退休人员基本养老金涨幅5.9%，月人均增加超过140元。民生补短板工程顺利推进，市图书馆新馆完成主体工程，市工人文化宫改扩建工程主体封顶，市体育馆完成主体混凝土结构施工，市博物馆和档案馆、市青少年宫和妇女儿童活动中心开工建设。坚持“房子是用来住的、不是用来炒的”定位，及时出台房地产调控措施，开展房地产领域专项治理。开工建设棚户区改造4069套，建成公租房4339套，全面完成目标任务。

全面加强社会治理。持续开展普法宣传教育，不断增强市民法治观念。扎实推进北海公安工作“六大工程”和“10+1+1”大会战，严厉打击违法犯罪活动。落实安全生产工作各项举措，没有发生重特大安全生产事故。扎实推进应急管理体系建设，建成市级应急管理平台，率先在全区建立海陆空一体应急志愿者队伍。骆春伟英雄事迹感动中国，成为干部群众学习的榜样和标杆。市公安局获公安系统全国精神文明建设先进单位，铁山港区获全国平安建设先进县区，银海区贵兴社区获全国扫黄打非进基层示范点和示范标兵荣誉称号。

外事、侨务、民族、宗教、地方志、档案、妇女儿童、残疾人、海防、人防、双拥、防震减灾等方面工作取得新成效。

（八）着力加强政府自身建设。坚持和加强党的全面领导，加快转变政府职能，着力建设法治政府和服务型政府。严格落实市委常委会“三重一大”集体决策制度，认真执行市人大及其常委会决议、决定，定期向市人大常委会报告工作、向市政协通报情况，主动接受市人大的法律监督、工作监督和市政协的民主监督，44件人大代表建议、351件政协提案全部办结，满意率达100%。深化“放管服”改革，不断优化政务环境，市政务服务中心被评为全国行政服务大厅优秀案例。深入推进党风廉政建设，加强审计工作，强化行政监察。持之以恒改进作风，大力践行“三坚持两改变”，扎实推进“两学一做”学习教育常态化制度化，深入开展机关作风建设“治庸提效”专项活动、提效能争先锋“十个一”活动，真抓实干成为新风尚。配合市人大开展地方性法规立法工作，颁布实施我市第一部实体性地方法规《北海市城市市容和环境卫生管理条例》。市长出庭应诉行政诉讼案开创广西先例。

各位代表！过去一年，我们紧抓机遇、勇于担当、砥砺前行，成绩来之不易，进步难能可贵。这是自治区党委、政府和市委统筹全局、正确领导的结果，是市人大及其常委会和市政协有效监督、大力支持的结果，也是全市人民同心同德、开拓进取的结果。在此，我谨代表市人民政府，向全市广大干部群众，向人大代表、政协委员，向各民主党派、各人民团体、各界人士和驻市单位、驻市部队、武警官兵，向所有关心支持北海建设发展的同志们、朋友们，表示衷心的感谢，致以崇高的敬意！

我们也清醒地认识到，北海经济社会发展和政府工作还存在不少困难和问题：一是实体经济下行压力仍然较大，固定资产投资增长缓慢，民间投资增长乏力；二是一些重点项目、重点工作推进不快，没能按照时间节点完成任务；三是民生领域还存在不少短板，教育、医疗的优质资源不足；四是城市基础设施欠账较多，参与“一带一路”建设的基础不够扎实，抓手较少；五是城市管理的精细化程度不高，水、大气等环境治理工作压力较大；六是政府系统少数干部担当意识和服务能力不强，行政效能仍有较大提升空间。我们将坚持问题导向，采取有针对性的措施，认真加以解决。

二、2018年工作总体要求和主要目标

党的十九大作出了中国特色社会主义进入新时代的重大判断，确立了习近平新时代中国特色社会主义思想的指导地位，开启了全面建设社会主义现代化国家新征程。市委十一届四次、五次全会认真贯彻落实党的十九大精神，在准确把握北海发展所处历史方位、科学分析北海发展阶段性特征的基础上，作出了建设富强、开放、生态、文化、幸福新北海的战略安排，对今年全市工作进行了全面部署。这些安排部署，是我们谋划政府工作、制定发展目标的重要遵循。

今年政府工作的总体要求是：坚持以习近平新时代中国特色社会主义思想为指导，全面贯彻落实党的十九大精神和习近平总书记视察广西及北海重要讲话精神，紧紧围绕自治区党委、政府和市委的决策部署，统筹推进“五位一体”总体布局和协调推进“四个全面”战略布局，坚持稳中求进工作总基调，坚定不移贯彻新发展理念，牢牢把握高质量发展根本要求，深化供给侧结构性改革，持续推进“五个扎实”，深入实施“四大会战”，奋力舞起“四个龙头”，开启新时代北海发展新征程，为在广西率先全面建成小康社会、加快建设创新型开放城市、力争建成广西副中心城市打下坚实基础。

今年全市经济社会发展的主要目标是：地区生产总值增长8.7%以上，财政收入增长8%以上，固定资产投资增长8%以上，规模以上工业增加值增长10.5%以上，外贸进出口总额增长8%以上，社会消费品零售总额增长9.5%以上，城镇居民、农村居民人均可支配收入增长8%以上，城镇新增就业人数2万人，城镇登记失业率控制在4%以下，居民消费价格指数控制在3%以内，全面完成自治区下达的能源消费总量及强度“双控”和控制温室气体排放任务。

做好今年政府工作，必须做到“六个坚持”。一是坚持以加强党的全面领导为根本聚合力。把全面从严治党作为政府工作的根本保证，切实增强“四个意识”，坚定“四个自信”，坚决执行中央、自治区和市委的决策部署，维护市委权威，提高政治能力，增强执政本领，团结一切力量，凝聚最大共识，形成推动北海发展的强大合力。二是坚持以新发展理念为统领明思路。发展是解决北海一切问题的基础和关键，要立足广西、放眼全国、走向世界，不断壮大经济实力，努力实现从高速增长阶段向高质量发展阶段转变，争当落实新发展理念、实现高质量发展的排头兵。三是坚持以打造向海经济为重点稳增长。北海向海而生、向海而兴，一定要挖掘潜力，用好资源，发挥优势，深度融入“一带一路”建设，加快发展外向型经济，在打造向海经济中培育新动能、催生新增长点，增强高质量发展的支撑。四是坚持以推进项目建设为抓手增后劲。牢固树立抓项目促发展的思想，加大招商引资力度，不断策划包装推出新项目，储备一批、建设一批、谋划一批，确保项目建设不断档、跟得上。持续优化投资和项目建设环境，为项目建设提供最优质服务、最坚强保障。五是坚持以加强环境保护为基础优生态。牢固树立绿色发展理念，以绿色经济为主攻方向，大力实施生态立市战略，实施最严格的生态环境保护，守住“绿水青山、碧海银滩”，把北海城乡建设得更宜居、更美丽。六是坚持以回应群众关切为突破惠民生。坚持以人民为中心的发展思想，民有所呼，政必有应，把群众所想所盼作为政府工作的着力点和立足点，持续不断每年解决一批群众要求强烈、期待迫切的问题，让群众有更多的获得感。

三、2018年重点工作任务

今年是全面贯彻落实党的十九大精神的开局之年，是决胜全面建成小康社会、实施“十三五”规划承上启下的关键一年，是改革开放四十周年、自治区成立六十周年、北部湾经济区发展规划上升为国家战略十周年，做好今年工作、推动北海高质量发展意义重大、任务艰巨。我们一定要善于通观全局、统筹兼顾，突出重点、把握关键，着重抓好以下十一个方面工作。

（一）加快打造向海经济。发挥区位和资源优势，做足、做深、做大海的文章，加快引进和建设一批向海经济的标志性基础设施和产业项目，把向海经济打造成为新时代北海高质量发展的主引擎和增长极。

科学谋划向海经济。坚持生态优先、陆海统筹，紧紧扭住绿色发展、枢纽建设、开放合作、海洋产业等重点，高水平谋划发展向海经济。深入实施《关于打造向海经济的行动方案》，高起点编制向海经济发展战略规划，科学布局重点产业、发展区域，加快形成规划有序、定位明确、特色鲜明的向海经济发展格局。

夯实向海经济支撑基础。大力推进交通、信息等基础设施建设，促进公路、铁路、港口和航空各种运输方式的有效衔接，推进口岸设施建设和功能升级，加快完善互联互通网络，增强城市的承载能力和辐射能力。公路方面，重点推进南北高速（钦州-山口段）改扩建、松旺至铁山港东岸高速公路（北海段）、北铁一级公路、广西滨海公路山口支线、大风江至牛尾岭段和关井至营盘段等项目建设。铁路方面，重点推进合湛铁路、铁山港至石头埠及啄罗支线、铁山港进港铁路等项目建设，争取启动建设北海北站综合交通枢纽工程，加快推进北海动车所、合浦至铁山港铁路、玉林至北海城际铁路、贵港至北海城际铁路等项目前期工作。港口码头方面，推进航道、泊位和港口配套设施建设，加快打造铁山港亿吨大港，推进铁山港航道疏浚三期工程、铁山港7-8#泊位工程、东港区榄根作业区1-2#泊位工程和铁山港公共执法码头、液体化工码头工程。航空方面，加快完善通达北海的“空中桥梁”网络体系，优化重点航班时刻，加快开通重点区域航线，开工北海机场停机坪扩建工程，力争今年北海机场旅客吞吐量突破200万人次。

大力发展海洋产业。以加快建设国家海洋经济创新发展示范城市为目标，以推进国家第四海洋研究所和国家科技兴海产业示范基地建设为抓手，加快转型升级传统海洋产业，培育发展海洋新兴产业。重点发展临海工业、海洋运输、海洋生物、海洋高端装备制造、远洋捕捞、海水养殖、海产品精深加工和仓储物流等产业，抓好渔港规划和升级改造、海洋牧场和银海蓝色产业园等建设。切实提高海洋科技创新能力，不断夯实海洋产业科技支撑。启动北海蓝碳试点申报工作。

全面振兴南珠产业。深入实施振兴南珠产业计划，加快编制“一区三中心”专项规划，科学确定南珠产业空间布局，保障养殖用海专区专用，加快建设南珠现代特色养殖核心示范区。加大财政对南珠产业的支持力度，健全南珠养殖、加工、产品、销售、管理的标准体系，延长和完善南珠产业链，推动南珠养殖、加工转型升级发展。挖掘南珠文化内涵，弘扬南珠文化，讲好南珠故事，扩大南珠品牌影响力，促进南珠产业与旅游产业融合发展。

（二）加快推动主导产业转型升级。深入实施工业转型升级大会战，深化供给侧结构性改革，推动电子信息、石油化工、临港新材料三大主导产业转型升级、补齐短板，培育实体经济发展新动能。

推动电子信息产业创新发展，力争完成产值1550亿元。重点推动惠科二、三期建设和8.6代薄膜晶体管液晶显示器件项目尽快落地，加快发展惠科移动智能终端产业集群；推进三诺倍增、冠捷提质扩能计划，加快中国-东盟信息港北海基地、中电北部湾信息港、电子信息物流平台、北部湾表面处理中心等平台建设，大力打造产业链完整的电子信息产业集群。

推动石油化工产业延伸发展，力争完成产值385亿元。提高北海炼化原油加工能力，加快推进新鑫能源30万吨劣质凝析油异构化、中航化20万吨液化气综合利用等一批配套项目，重点推进200万吨/年劣质重油深加工项目落地。

推动临港新材料产业提质增效，力争完成产值500亿元。开工建设总投资百亿元的信义产业园，加快推进诚德集团产能“填平补齐”工程，加快建设铁山港临港新材料产业园，推动一批新材料签约项目尽快落地，加大引进下游不锈钢产业项目力度。

推动林纸产业发展壮大，重点推进北部湾（合浦）林产循环经济产业园建设，引进一批林产项目。

（三）加快发展现代服务业。全面推进创建国家全域旅游示范区工作和全域旅游城市建设大会战，实施《北海市全域旅游发展总体规划》和三年行动计划。重点推进银滩改造提升和涠洲岛整体开发建设，力争涠洲岛南湾鳄鱼山创国家5A级旅游景区尽快通过国家评定，加快建设中信国安水乐园等主题游乐项目，完善提升北海老城、园博园等景区，统筹推进合浦汉文化公园、星岛湖、高德老街、白龙珍珠古城旅游区等景区景点开发。加快打造北部湾邮轮母港服务业集聚区，积极推进一批国际品牌高星级旅游酒店、特色主题公园和特色旅游小镇建设，建设完善观景、休憩、标识及吃住行游购娱等服务设施，规划建设游客集散中心，深入推进旅游厕所革命，建立全域旅游大数据中心。加强旅游市场监管，优化旅游环境。

加快提升商贸等服务业，支持商贸企业做大做强，挖掘批零住餐行业发展潜力，加强商贸企业入统工作。大力推广电子商务应用，拓展农村电商、旅游电商、跨境电商等领域。加快发展软件和信息服务业，积极发展港航物流、文化创意等服务业。着力防范化解重点领域风险。加大“引金入北”工作力度，大力发展金融服务业，营造良好的金融生态，充分发挥金融服务实体经济的作用。加强房地产市场预测和监管，更好地维护市场稳定、防范风险。

（四）加快推进项目建设扩大有效投资。发展是第一要务，项目是第一载体，有大项目才有大发展。要以“踏石留印、抓铁有痕”的劲头，大力开展“投资促进年”活动，强力推进项目建设，切实扩大有效投资，促进经济高质量发展。

大力开展招商引资。坚持把招商引资作为经济建设的“第一抓手”，持之以恒抓好招大引强工作。开展招商资源普查，创新招商引资方式，强化精准招商、产业链招商、以商招商，紧紧围绕三大主导产业和海洋经济、全域旅游、林纸一体化等产业开展招商工作，积极争取区直部门的基金、直投、债券、贴息的支持，让更多的新项目、大项目落户北海。

大力推进项目建设。继续强化全市重大项目策划与推进工作机制，采取“四定”办法，加强项目对接与协调,保障重大项目土地、资金等要素需求,及时解决项目建设过程中遇到的困难和问题。筹划和储备一批带动性强、关联度大、发展前景好的项目。大力推进涠洲岛、银滩、邮轮母港、合浦海上丝绸之路等四个百亿级项目集群，推动水上飞机等一批重点项目尽快开工建设，不断掀起项目引进建设的热潮。

大力优化营商环境。既要继续改善硬环境，更要在软环境建设上取得突破。大力深化“放管服”改革，推进政务服务事项线上线下一体化办理，提供便捷的在线咨询、申请、查询、投诉等服务。进一步优化审批流程，压缩办理时间，拓宽重大项目行政审批“绿色通道”。推进信息共享、照证一窗通，推行行政审批容缺办理承诺制。加强审管联动和事中事后监管。实施市场准入负面清单制度，保障不同所有制企业公平待遇。继续完善“单一窗口”建设，提高通关便利化水平。

（五）加快推进城市规划建设管理。深入实施城乡建设管理大会战，在“城乡环境提升年”取得明显成效的基础上，保持频道不换、力度不减，深化城乡环境“十大提升行动”，推动城市环境综合整治取得新成效，进一步完善城市功能、提升城市形象。

提升城乡规划设计。重点推进城市设计试点工作，突出北海阳光、开放、明快的滨海城市特色，按照控规+城市设计的思路推进控规全覆盖，完善城乡规划管理技术规定，落实海绵城市、绿色建筑、开放式街区等要求，高水平开展总体城市设计。

深化城市环境整治。聚焦群众高度关注的问题，着手除痛点、解难点、减热点、增亮点，重点整治市容环境卫生、交通秩序，抓好“城中村”改造、市政园林建设、违法建设整治、市场改造提升和小区管理等工作。加快实施滨海国家湿地公园（冯家江流域）水环境治理、垃圾焚烧发电、城市污水治理、银滩东区大冠沙部分市政基础设施路网等PPP项目。

推进市政基础设施建设。以创建宜居城市为目标，优化城区交通路网，拓展城市发展空间。开工建设西村港大桥，加快西南大道东延线建设，完成南珠大道改造等对外连接道路工程。完善铁路以南“小三纵三横”主干路网和银滩沿线市政路网，加快建设铁路以北湖海路、重庆路、湖北路等道路，启动建设环北海半岛最美滨海骑行线。推进海洋产业科技园创业谷湿地长廊等海绵城市示范项目，完成云南路雨水支渠、南京路雨水排放口等排水工程。实施城市绿化、美化和亮化行动，继续推进黄金北岸、海丝路广场、铁路东段绿廊建设。加快建设城市配电网和通信基础设施，完成国家电信普惠试点城市建设。

加快推进场馆建设。建成市图书馆新馆、市体育馆、市老干部活动中心，中山公园、长青公园升级改造竣工开园，推进市博物馆和档案馆、市青少年宫和妇女儿童活动中心等场馆建设，完善西区儿童公园设施，开工建设南珠市民休闲广场，加快市群艺馆迁建前期工作。

提高城市治理水平。充分运用“共同缔造”理念，动员各界力量，推动城市建设决策共谋、发展共建、建设共管、效果共评、成果共享。加快建设智慧城市，初步建成“数字城管”，推进城市管理精细化、信息化、网络化、智能化，打造市区严管街、示范路，继续推行小广告清理、卫生保洁工作市场化。加强城市交通秩序管理，大力推行机动车“礼让斑马线”。

（六）加快建设生态美丽北海。生态是北海的生命。要践行绿水青山就是金山银山的理念，深入实施生态立市战略，坚持生态优先、绿色发展。

划定生态红线，严守开发边界，加强自然保护区、湿地公园和森林公园的建设管理，严厉打击各种破坏生态环境行为。切实加强永久基本农田保护，守住耕地保护红线。坚决打赢污染防治和水环境治理攻坚战，全面推行“河长制”，大力推进南流江、冯家江、西门江等流域环境综合整治，重点加强近岸海域污染防治、入海排污口及黑臭水体专项整治。大力推动牛尾岭水库水源地环境治理、洪潮江第二水源地项目和禾塘水厂水井替代项目建设。完善城市雨污分流设施，加快推进餐厨垃圾处理、乡镇污水处理厂等污垃设施建设。加大城市扬尘治理力度，严控建筑工地扬尘、工业企业废气排放、车辆尾气排放和随意焚烧垃圾、焚烧秸秆，坚决守住“北海蓝”。大力发展生态经济，推动产业绿色转型优化，倡导简约、绿色、低碳的生活方式。

（七）加快实施乡村振兴战略。坚持农业农村优先发展，围绕产业兴旺、生态宜居、乡风文明、治理有效、生活富裕的总要求，制定实施乡村振兴规划，设立乡村振兴专项资金，扎实推进乡村建设各项工作。

坚决打赢脱贫攻坚战。深入实施农村脱贫攻坚大会战，在提高精准脱贫质量上下功夫，做到脱真贫、真脱贫。加大资金整合投入，加大到户特色产业扶持，提升产业增收对脱贫的贡献率。大力扶持发展贫困村集体经济，壮大村民合作社。强化以“一帮一联”为重点的结对帮扶，更加注重扶贫同扶志、扶智相结合。加强对扶贫资金使用的监督管理，强化扶贫领域执纪监督问责。按照“十一有一低于”和“八有一超”的标准，落实“三员九有”要求，打好精准扶贫“组合拳”，确保到2018年底全市79个贫困村和4.6万农村贫困人口达到脱贫摘帽标准，贫困人口年人均纯收入超过当年的国家扶贫标准。

大力发展现代农业。深入推进农业供给侧结构性改革，发展高端高质高效农业，切实提高农民收入。全面启动现代特色农业示范区增点扩面提质升级三年行动计划，推进1.5万亩“双高”糖料蔗基地建设。加快发展“一村一品”“一乡一业”优势特色农业产业，积极培育农民专业合作社、家庭农场、农业龙头企业等农业经营主体。加快实施台湾农渔业合作系列项目。不断完善“互联网+农业”发展模式，拓宽延长农业产业链，推进农村一二三产业融合发展。加强农产品质量安全监管和农产品品牌“三品一标”认证监管，保障优质安全的农产品供给。

发展壮大县域经济。统筹谋划县区发展，完善支持县区发展的政策措施，推动县域经济转型升级。加快推进市区与合浦县城一体化。大力发展合浦工业，加快合浦工业园区、铁山东港产业园开发建设，完善产业转移园、儒春田园综合体基础设施，引进特色产业项目。充分挖掘文化旅游的潜力，推进汉文化考古遗址公园等重点旅游项目建设，加快创建广西特色旅游名县和全域旅游示范区。大力发展铁山港区临港工业，加快推进铁山港新城规划建设，实现产、港、城良性互动发展。实施乡镇提升三年行动计划，加快打造“九大特色小镇”。

扎实推进宜居乡村建设。大力实施产业富民十项进村行动，加强农村基础设施和公共服务设施建设，推进“三改七提三增”工程，改善农村群众生产生活条件。抓好新时代讲习所建设，打造新型农民队伍，提高干部群众的思想道德素质、科技文化水平和生产技能。

（八）加快深化改革扩大开放。推进以科技创新为核心的全面创新，深化重点领域改革，主动融入“一带一路”建设，增创北海发展的新动能、新空间。

深入实施创新驱动发展战略。把创新摆在发展全局的核心位置，进一步落实创新驱动发展各项计划、规划和配套政策措施，增强科技创新在主导产业、海洋产业、战略性新兴产业、现代农业中的支撑能力。强化企业创新主体地位，大力培育创新型企业，鼓励企业持续加大研发投入和人才引进，推动创新资源向企业聚集，促进产学研一体化协同发展。弘扬“工匠精神”，深入推进质量强市，积极实施品牌战略。加强创新平台载体建设，积极打造自主创新示范区、海洋科技创新示范集聚区等一批创新平台。完善创新创业政策和服务保障体系，加强知识产权创造、保护和运用，设立科技成果转化基金，促进科技成果转移转化，引进和培养创新创业人才，推动科技与经济深度融合，着力打造北部湾创新高地。

深入推进重点领域改革。尽快理顺银滩旅游度假区、铁山港临海工业区和市四大国有投融资平台管理体制，推动市融资平台公司转型发展。全面完成农村土地承包经营权确权登记颁证工作，推进县区农村产权流转交易中心建设。积极申报国家级旅游业改革创新示范区,推动涠洲岛旅游区“多规合一”“两违”防控治理、新能源汽车推广应用等改革试点。深化公立医院综合改革，落实县区财政体制改革新措施，统筹推进投融资、金融改革，加快推进文化、教育和社会保险等领域改革。

深入实施开放带动战略。加快开放发展步伐，积极融入“渝桂新南向通道”建设，推进向东融通，积极对接“粤港澳大湾区”建设。拓宽利用外资渠道，引进更多外资项目，提升利用外资水平。大力发展对外贸易，深入实施加工贸易倍增计划，积极发展跨境贸易和服务贸易，扎实推进CEPA服务贸易先行先试。深化与“一带一路”沿线国家和地区开放合作，积极融入中国—东盟自贸区升级版建设，拓展与北部湾城市群区域合作，扩大与粤港澳台等地区的经贸往来，主动承接产业转移。扎实推进海洋合作试验区和旅游合作区建设，加快建设桂台产业合作北海示范区、龙港新区及玉港合作园。完善产业园区基础设施，促进园区升级发展，力争北海工业园区升级为国家级经济技术开发区。集中力量举办“魅力之城”系列活动和“北海国际珍珠节”。

（九）加快推动文化繁荣兴盛。增强文化自觉，坚定文化自信，加强海上丝绸之路文化遗产保护与利用，提升文化软实力。

培育和践行社会主义核心价值观。加快实施公民道德建设工程，把社会主义核心价值观融入到北海建设与发展各方面，让崇德向善成为北海人民的自觉追求。全市动员、全民参与，持之以恒开展创建文明城市活动，提升市民文明素质和城市文明程度。

积极发展文化事业。支持文艺创作与繁荣，培养和引进艺术门类的领军人才，打造一批富有北海特色的文艺精品。进一步加快公共文化服务体系建设，实施文化惠民工程，丰富群众文化生活，繁荣北海主流文化。加强文物和非物质文化遗产的保护利用，大力推进海丝路申遗工作，持续开展与东盟各国人文交流。广泛开展全民健身活动，积极举办国际性、全国性体育赛事，提高北海举办体育赛事的能力和水平，打造活力北海。

鼓励发展文化产业。扶持发展水彩画、贝雕、角雕、木雕和民间收藏等传统工艺美术产业，支持拍摄《海上丝路南珠宝宝》续集，加快培育影视文化、创意设计和动漫等新型文化业态。

（十）加快发展社会事业。坚持以人民为中心的发展思想，突出问题导向，尽力而为、积极作为，切实保障和改善民生，不断满足人民群众日益增长的美好生活需要。

优先发展教育事业。巩固义务教育均衡发展成果，力争所有县区通过义务教育均衡发展国家评估。实施普及高中阶段教育攻坚计划，着力提升教育质量。加快发展现代职业教育，深化产教融合、校企合作。积极办好学前教育、特殊教育和网络教育。全面发展素质教育，实施“童子功1+N”计划。着力解决中小学课外负担重、择校热、大班额等突出问题。力促北航与自治区达成北航北海校区办学意向，建成北京八中北海分校新校区。深入实施名校长、名师、名班主任培养工程和教育系统引进优秀人才千人计划。推进教育信息化建设，加快建设教育人才公寓，支持和规范社会力量兴办教育。

加快建设健康北海。全面推进医疗联合体建设，重点构建紧密型医疗联合体。进一步落实家庭医生签约服务工作。实施名医工程，培养和引进一批高层次医疗卫生人才，打造医院品牌与特色专科，提高医疗质量和服务水平。大力推进一批重大医疗卫生项目建设，引进优质医疗资源，促进优质资源有序下放基层，加快解决看病难、看病贵问题。深入开展爱国卫生运动，扎实推进新一轮防艾攻坚专项工程，开展精神卫生综合管理、结核病分级诊疗和综合防治服务模式试点工作。鼓励开办中医诊所，提高中医药服务能力和传承创新。加快推进计划生育服务管理改革，提升妇幼健康管理水平。力争成功创建广西食品安全示范城市。鼓励社会资本进入健康养老、医疗领域。

努力完善社会保障体系。坚持就业优先战略和积极就业政策，完善公共就业服务，促进高校毕业生、城镇就业困难人员和农村转移劳动力就业创业。全面建成多层次社会保障体系，实施全民参保计划，完善城镇职工和城乡居民基本养老保险制度，完善城乡居民基本医疗保险制度和大病保险制度。完善城乡社会救助体系，落实最低生活保障制度，迁建北海福利医院、改造养老福利苑。创新方式方法，大力推进棚户区（危旧房）改造项目，加快推进公租房分配入住。

切实加强和创新社会治理。扎实开展“七五”普法宣传教育，落实各项法治建设任务，全面推进公共法律服务体系建设。强化社区、乡村组织自治功能，形成共建共治共享社会治理新格局。加快建设社会治安防控体系，推进平安北海（三期）建设，依法打击和惩治违法犯罪行为，营造良好的社会治安环境。保持高压态势打击传销，广泛发动群众参与，大力创建“无传销小区”和“无传销城市”。建立健全预防和化解社会矛盾机制，切实做好信访维稳工作，妥善处理信访突出问题。严格落实安全生产责任制，深化重点行业领域安全专项整治，有效遏制重特大安全生产事故发生。建立应急处理指挥平台，加强应急管理，提高防灾救灾减灾能力。

（十一）加快推进为民办实事项目建设。想群众所想、急群众所急、解群众所困，千方百计为群众办好十件实事，项目总投资41.5亿元，2018年内计划完成投资18亿元。

一是开工建设北海市滨海国家湿地公园（冯家江流域）水环境治理工程。

二是整治完成30条城区小街小巷，建成乡村硬化道路210公里。

三是建成通车高德旧路改造、拉萨路、残疾人康复综合服务中心门前规划路、侨景路、长沙路、杭州路、南珠汽车站北侧规划路、天府路、江苏路等9条市政道路。

四是开工建设一批城市停车设施，新增停车位3000个。

五是开工建设北海市第十二中学，建成海城区第二十小学主体工程，建成桂林电子科技大学北海校区附属幼儿园、银海区平阳镇中心幼儿园、银海区银滩镇中心幼儿园等公办幼儿园。

六是建成并投入使用合浦县第五人民医院门诊住院综合大楼，建成合浦县人民医院住院综合楼，完成合浦县红十字会医院住院综合楼主体工程。

七是开工建设地表水供水工程，包括建设北海市北郊水厂水质提标工程、牛尾岭水库水质修复工程等，年底实现地表水源和地下水源共同为城市供水的目标。

八是开工建设银滩西区健康绿色步道。

九是开工建设市兰花公园和一批城市街头公共绿地。

十是按照“六个一”标准基本建成341个行政村的村级公共服务中心。

四、切实加强政府自身建设

新时代要有新作为，新征程要有新担当。我们要坚持和加强党的全面领导，以提高政府执行力为主线，转作风、优服务、提效能，全面履职尽责，持续推进人民满意的法治政府和服务型政府建设。

进一步改进工作作风。巩固“两学一做”学习教育成果，抓好即将开展的“不忘初心、牢记使命”主题教育，努力践行“三坚持两改变”，推动干部作风持续好转。深入开展“治庸提效”活动，强化督查问责，严肃整治庸政懒政怠政行为，着力解决基层群众和投资者反映强烈的问题。建立健全激励机制和容错纠错机制，激发干部干事创业的热情。

进一步提高政府执行力。坚持把狠抓落实、有效执行作为政府工作的生命线，切实提高各级各部门行政效能。加快转变政府职能，提高工作效率,落实工作责任，营造良好的政务服务环境。大力推进“互联网+政务服务”建设，让企业和群众少跑腿、好办事、不添堵。倡导“马上就办”工作理念，用钉钉子的韧劲和言出必行的干劲，狠抓重点工作和重大项目推进落实，确保各项政策措施落地生根。

进一步严格依法行政。把政府工作全面纳入法治轨道，依法行使政府权力，严格规范公正文明执法，加强行政监督和审计监督。提高行政决策效率和质量，确保行政决策科学、民主、合法。配合市人大开展好涠洲岛、红树林、沿海沙滩、老街和合浦汉墓群等“五个保护条例立法”工作。依法接受市人大及其常委会的法律监督、工作监督，执行市人大及其常委会的决定、决议，支持人民政协履行政治协商、民主监督、参政议政职能，做好人大代表建议和政协提案办理工作。

进一步加强廉政建设。严格落实全面从严治党主体责任，持续提升政府系统从严管党治党水平。严守政治纪律和政治规矩，严肃党内政治生活，强化党内监督，严格执行中央八项规定实施细则，聚焦形式主义、官僚主义十种新表现，坚持纠正“四风”不止步，充分运用监督执纪“四种形态”，抓早抓小、防微杜渐，把党风廉政建设和反腐败斗争引向深入,大力营造风清气正的政治生态。

各位代表！

新的时代鼓舞人心，新的征程催人奋进。让我们更加紧密地团结在以习近平同志为核心的党中央周围，在自治区党委、政府和市委的坚强领导下，不忘初心，牢记使命，乘势而上，努力以新作为开启新征程、以新担当推动新发展，奋力谱写新时代北海发展新篇章！
